# Intrabody Tetrodotoxin Distribution and Possible Hypothesis for Its Migration in Ribbon Worms *Cephalothrix* cf. *simula* (Palaeonemertea, Nemertea)

**DOI:** 10.3390/md19090494

**Published:** 2021-08-29

**Authors:** Grigorii V. Malykin, Alexei V. Chernyshev, Timur Yu. Magarlamov

**Affiliations:** A.V. Zhirmunsky National Scientific Center of Marine Biology, Far Eastern Branch, Russian Academy of Sciences, 690041 Vladivostok, Russia; gmalykin@imb.dvo.ru (G.V.M.); achernyshev@imb.dvo.ru (A.V.C.)

**Keywords:** tetrodotoxin, TTX, TTX distribution, CLSM, LC–MC/MC, ribbon worm, Nemertea, *Cephalothrix*, toxin migration

## Abstract

Tetrodotoxin (TTX) is a potent neurotoxin found in many marine and terrestrial animals, but only a few species, such as the ribbon worms of the genus *Cephalothrix*, accumulate it in extremely high concentrations. The intrabody distribution of TTX in highly toxic organisms is of great interest because it helps researchers to understand the pathways by which the toxin migrates, accumulates, and functions in tissues. Using immunohistochemistry with anti-TTX antibodies, the authors of this study investigated the toxin’s distribution inside the organs, tissues, and cells of *Cephalothrix* cf. *simula*. The cell types of TTX-positive tissues were identified by light microscopy. The main sites of TTX accumulation occurred in the secretory cells of the integuments, the microvilli of the epidermal ciliary cells, cephalic glands, the glandular epithelia of the proboscises, the enterocytes of the digestive systems, and nephridia. Obtained data suggest the toxin migrates from the digestive system through blood vessels to target organs. TTX is excreted from the body through the nephridia and mucus of epidermal cells.

## 1. Introduction

Nemertea (ribbon worms) comprise a phylum of invertebrates, mainly marine animals, that are currently divided into three classes: Palaeonemertea, Pilidiophora, and Hoplonemertea [1]. Most nemerteans are active predators but also feed on carrion [2,3,4]. Small crustaceans, polychaetes, and mollusks are high on the list of food priorities. Even though all the members of the phylum are soft-bodied (i.e., have no external mechanically protective structures), they do not usually have natural predators. For hunting and protection, nemerteans secrete a poison containing a wide range of toxins [5,6]. The only toxin found in representatives of all three classes is tetrodotoxin (TTX), which was discovered more than 30 years ago [7]. In subsequent years, the presence of TTX and its analogues (TTXs) in nemerteans was reported by various scientific groups [8,9,10,11], but the genus *Cephalothrix* was found to have a toxin concentration comparable to that of the pufferfish and blue-ringed octopuses [8,10,11,12,13,14,15], making it the only nemertean species to be classified as “extremely toxic” (toxicity higher than 1000 MU/g (178 μg/g)), according to the toxicity classification established by Japan’s Ministry of Health, Labor and Social Security [10].

In 2004, Tanu et al. [16] were the first to investigate the intrabody distribution of TTX in an unidentified species of the genus *Cephalothrix*, but they only studied the intrabody distribution in the foregut region and only described TTX-positive cells, not all the cell types in TTX-positive tissues and organs. In the current work, four regions of toxic *Cephalothrix* cf. *simula* were studied: the precerebral, the foregut, and the anterior and posterior regions of the intestine. At the light-optical level, all the cell types of TTX-positive tissues (the cephalic gland, the integument, the epithelium of digestive system, the glandular epithelium of the proboscis, and the blood vascular and excretory systems) were characterized. Using confocal laser-scanning microscopy (CLSM) with anti-TTX antibodies and cryosections, the types of cell accumulating the toxins were identified, thereby providing new data on toxin localization. Issues regarding toxin intake and migration within the nemertean’s body, as well as TTX functions, are discussed.

## 2. Results

### 2.1. TTX Measurement by LC–MS/MS

**Figure 1:**
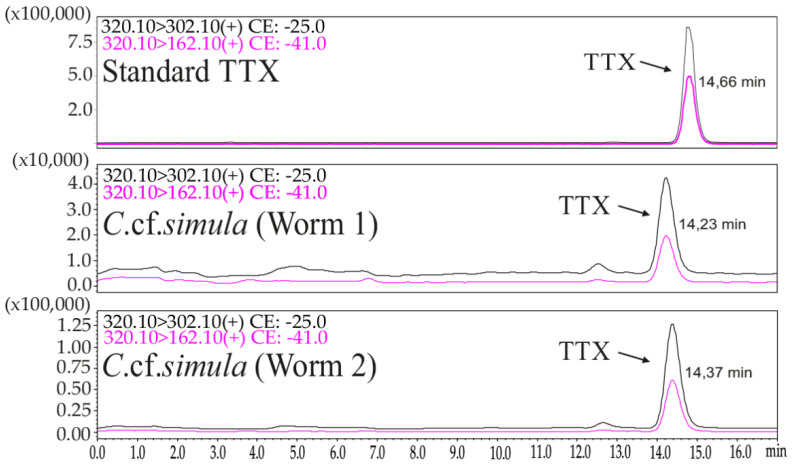
High-performance liquid chromatography–tandem mass spectrometry (HPLC–MS/MS) chromatograms of standard tetrodotoxin (TTX) and TTX obtained from whole-body extracts of two worms of *Cephalothrix* cf. *simula*.

High-performance liquid chromatography–tandem mass spectrometry (HPLC–MS/MS) analysis revealed TTX in whole-body extracts of two specimens of *Cephalothrix* cf. *simula*: in Worm 1, the TTX concentration was 13,478.26 ng/g of body weight, and in Worm 2, it was 9685 ng/g of body weight (Figure 1).

### 2.2. TTX Distribution by CLSM

**Figure 2:**
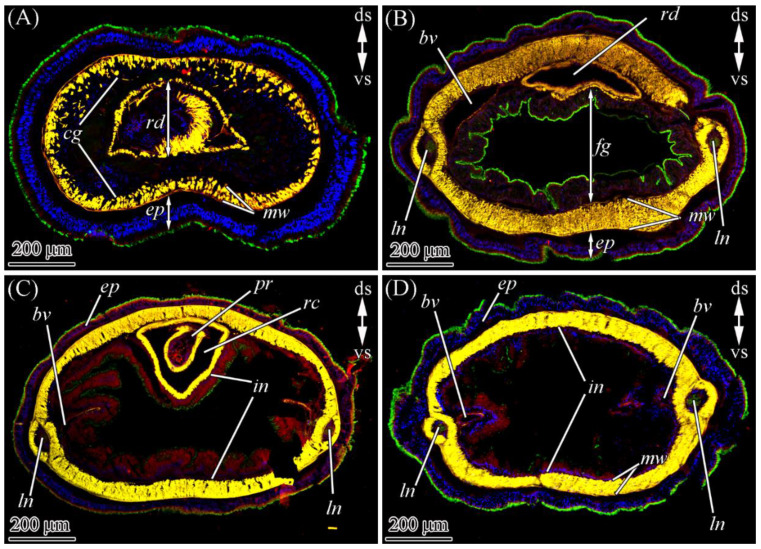
Tetrodotoxin (TTX)-like immunoreactivity in different body regions of *Cephalothrix* cf. *simula*. The confocal laser scanning micrographs show substacks of transverse sections. Red, TTX-like immunoreactivity; green, α-acetylated tubulin immunoreactivity; blue, nuclei, (4′,6-diamidino-2-phenylindole (DAPI) stain; yellow, musculature, phalloidin-positive. (**A**) Precerebral region. (**B**) Foregut region. (**C**) Anterior intestine region. (**D**) Posterior intestine region. bv, blood vessel; cg, cephalic gland; ds, dorsal side; ep, epidermis; fg, foregut; in, intestine; ln, lateral nerve; mw, musculature of body wall; pr, proboscis; rc, rhynchocoel; rd, rhynchodaeum; vs, ventral side.

**Figure 3:**
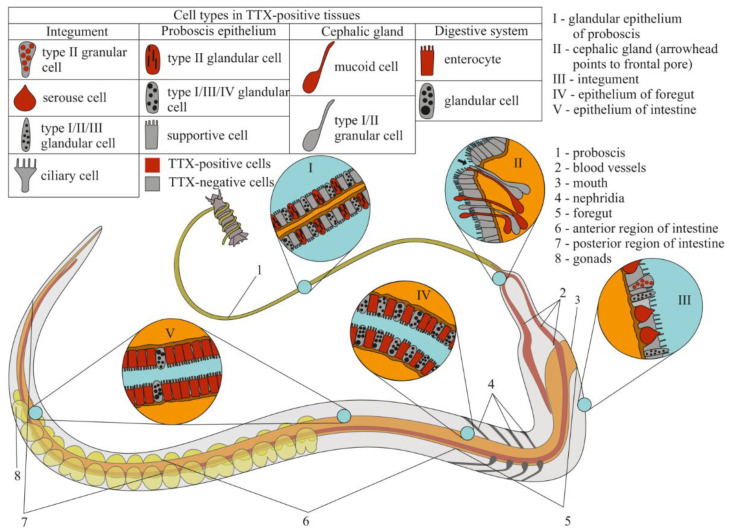
Schematic illustration of tetrodotoxin (TTX) distribution in the proboscis, cephalic gland, integument, and digestive system of *Cephalothrix* cf. *simula*.

CLSM with the anti-TTX antibodies revealed the toxin in all four regions (the precerebral region, the foregut, and the anterior and posterior regions of the intestine) of two worms (Figure 2). An intense label was found in the glandular epithelium of the proboscis, nephridia, epidermis, foregut, and intestinal epithelia (Table 1 and Figure 3). A medium-intensity label was detected in the cephalic gland, lateral nerves, and oocytes (Table 1). Weak TTX-like immunoreactivity was observed in the musculature of the body wall and proboscis, as well as in the endothelium of blood vessels (Table 1).

#### 2.2.1. Glandular Epithelium of the Proboscis

**Figure 4:**
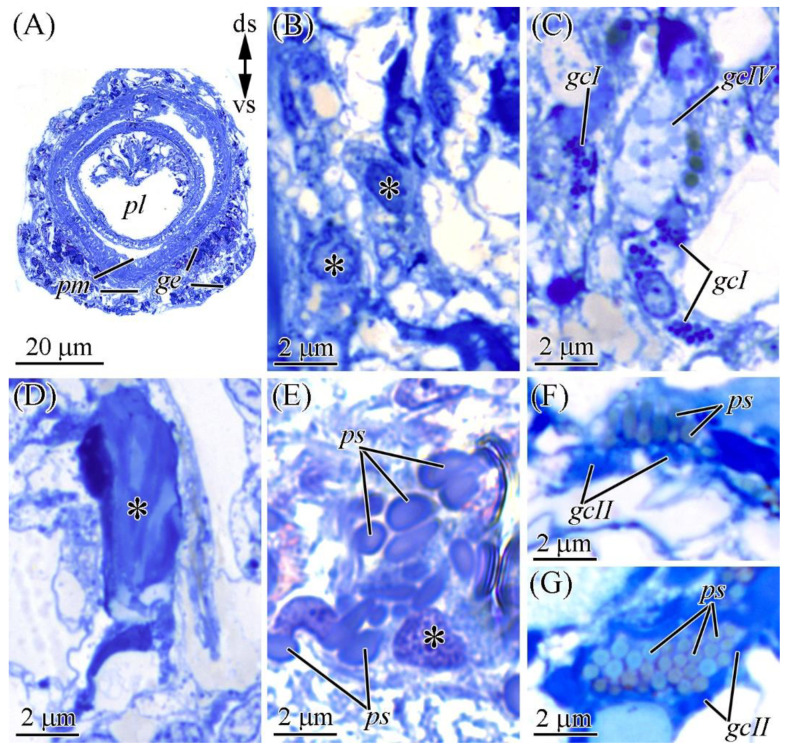
Light micrographs of the transversal (**A**–**F**) and longitudinal (**G**) sections of the proboscis of *Cephalothrix* cf. *simula*. (**A**) Panoramic view of the partially everted proboscis. (**B**) Supportive cells (asterisks). (**C**) Type I (gcI) and VI (gcIV) glandular cells. (**D**) Type II gland cell (asterisk). (**E**) Pseudocnidae-containing cell (asterisk) with pseudocnidae (ps). (**F**) Pseudocnidae released on the apical surface of proboscis epithelium. (**G**) Apically situated pseudocnidae surrounded by type II gland cells (gcII). ds, dorsal side; gcI, type I gland cell; gcII, type II gland cell; gcIV, type IV gland cell; ge, glandular epithelium of proboscis; pl, lumen of inverted proboscis; pm, proboscis musculature; ps, pseudocnidae; vs, ventral side.

The glandular epithelium of the proboscis consists of supportive cells and four types of glandular cells (Figure 4A). Supportive cells are slender, with distally branched extensions terminating at the epithelium surface (Figure 4B). Type I glandular cells (see [17]) are typically oval-shaped (Figure 4C) and filled with spherical granules up to 0.4 μm in diameter, and type II or bacillary glandular cells (see [17]) are oval with a slightly rounded base. The entire free internal space of these cells is filled with rod-shaped (bacillary) secretory granules (Figure 4D). Type III, or pseudocnidae-containing, cells contain pseudocnidae—unique to nemerteans structures that, like the cnidae of Cnidaria, discharge an inner filament (core) (see [18]) (Figure 4E). Mature pseudocnidae released onto the apical surface of bacillary cells form monolayered clusters (Figure 4F,G). Type IV glandular cells (mucoid glandular cells; see [17]) are oval (Figure 4D), and their middle and apical regions are filled with rounded granules (about 0.6 µm in diameter).

**Figure 5:**
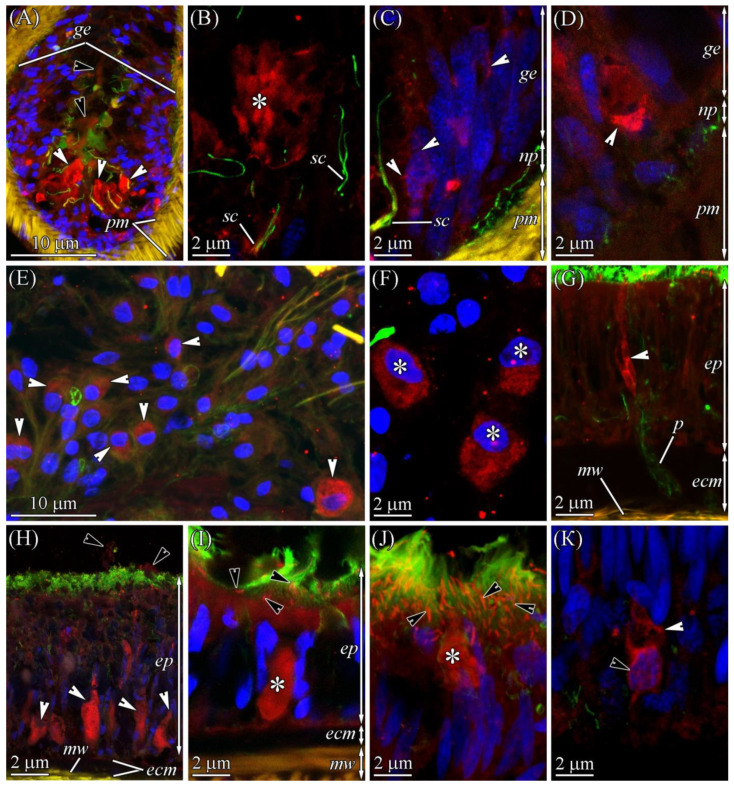
TTX-like immunoreactivity of the everted proboscis (**A**–**D**), cephalic gland (**E**–**G**), and integument (**H**–**K**) of *Cephalothrix* cf. *simula*. The confocal laser scanning micrographs show substacks of transverse sections. Red, TTX-like immunoreactivity; green, α-acetylated tubulin immunoreactivity; blue, nuclei (DAPI); yellow, musculature, phalloidin-positive. (**A**) Panoramic view of proboscis; white arrowheads point to TTX-positive cells, and black arrowheads point to TTX-positive slime on the apical surface of the glandular epithelium and in the proboscis lumen. (**B**) TTX-positive granules of type II gland cells (asterisk). (**C**) The bodies of cells with TTX-positive structures; arrowheads point to elongated sickle-shaped structures. (**D**) TTX-positive sickle-shaped structure of the epithelial cell. (**E**) Cephalic gland with TTX-positive mucoid cells (arrowheads). (**F**) TTX-positive cell bodies of mucoid cells (asterisks). (**G**) TTX-positive ducts/lacunar extensions (arrowhead) of the mucoid cell passing through the pore of extracellular matrix (ecm) and integument. (**H**) Panoramic view showing TTX-positive granules of serous cells (white arrowheads) and TTX-positive secretions (black arrowheads) onto the surface of the epidermis (ep). (**I**) The TTX-positive granule of the serous cell (asterisk) and TTX-positive microvilli of ciliary cells (arrowheads). (**J**) The distal region of the epidermis with apical extension (asterisk) filled by TTX-positive spherical granules. Arrowheads point to the TTX-positive microvilli of ciliary cells. (**K**) The proximal region of the epidermis with TTX-positive filamentous structures surrounding the nucleus (black arrowhead) and occupying the perinuclear region (white arrowhead). ecm, extracellular matrix; ep, epidermis; ge, glandular epithelium of proboscis; mw, musculature of body wall; np, basiepithelial nerve plexus; p, pore; pm, proboscis musculature; sc, sensory cell.

The CLSM images showed that in the proboscis, TTX localized in type II glandular cells, on the surface of the glandular epithelium, and in the proboscis lumen (Figure 2C and Figure 5B; Table 1). TTX-positive secretion onto the cell surface and inside the lumen was diffuse, with low intensity. The secretory granules of type II glandular cells exhibited high-intensity TTX-labeling. Z-projections revealed that the TTX labeling in some granules was weak or absent, which was possibly due to the artifacts of sample preparation. TTX-positive structures with middle-intensity labeling were revealed in the bodies of epithelial cells (Figure 5C,D). These structures were either elongated, sickle-shaped or thin filaments. The sickle-shaped structures predominantly localized in the distal part of the cell body (Figure 5D), while filamentous structures formed a net surrounding the nucleus and occupying the entire perinuclear region (Figure 5C). Presumably, the TTX-positive structures in the bodies of the cells represent the membrane-enclosed organelles of protein secretion (the nuclear envelope, endoplasmic reticulum membrane, and Golgi complex), which is supported by immuno-electron investigations of heteronemertean *Kulikovia alborostrata* (also known as *Lineus alborostratus*) (see [9]). It was not possible to determine to what type of cells the TTX-positive bodies belonged.

#### 2.2.2. Cephalic Gland

**Figure 6:**
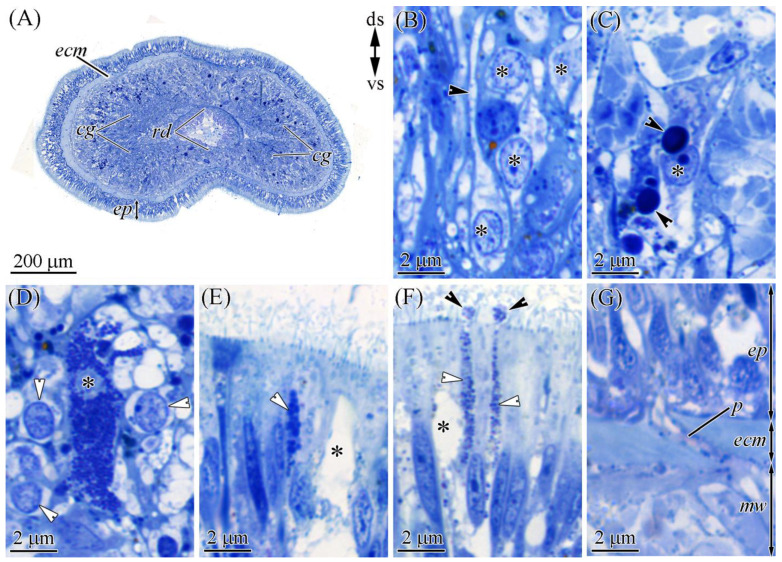
Light micrographs of the transverse sections of the precerebral region of *Cephalothrix* cf. *simula*. (**A**) Panoramic view of the precerebral region. (**B**) Bodies of mucoid cells (asterisks). Arrowhead points to the duct of the mucoid cell. (**C**) The body of type I granular cell (asterisk). Arrowheads point to secretory granules. (**D**) Bodies of type II granular cells (asterisk) and mucoid cells (arrowhead). (**E**) Ducts of type I granular cell (arrowhead) and mucoid cell (asterisk). (**F**) Ducts of type I granular cell (white arrowheads) and mucoid cell (asterisk). Black arrowheads point to the papillae of type I granular cells. (**G**) Pore in the epidermal extracellular matrix. cg, cephalic gland; ds, dorsal side; ecm, extracellular matrix; ep, epidermis; mw, musculature of body wall; p, pore; rd, rhynchodaeum; vs, ventral side.

The cephalic gland occupies the precerebral region of *Cephalothrix* (Figure 6A) and, according to Ferraris [19], is represented by eight lobes (four on either side of the midline). Here, three types of glandular cells scattered among the cephalic gland were detected: mucoid (Figure 6B), type I (Figure 6C), and type II (Figure 6D) granular cells. Each of the lobes of the cephalic gland had all three types of glands. Each one formed a single extension (duct) that discharged either into the main channel of the cephalic lacuna (see Figure 4 in [19]) or singly (“lacunar extensions”; see [19]) along the entire length of the cephalic region. The lacunar extensions (ducts) passed through the pore in the epidermal extracellular matrix (ECM) and protruded into the epithelial surface, forming a papilla (Figure 6E,F). Mucoid cells were the main type of cell in the cephalic gland. The bodies of these cells were oval (about 3 µm in diameter) (Figure 6B,D). The ducts of these cells were 2–3 µm wide and filled with large secretory granules, the contents of which were not stained with methylene blue (Figure 6B). Type I granular cells had irregularly shaped bodies (about 4 µm in diameter) (Figure 6C). The perinuclear cytoplasm contained 1–3 oval secretory granules up to 1 µm in diameter. The ducts of these glandular cells were filled with spherical secretory granules (about 0.3 µm in diameter) (Figure 6D). Type 2 granular cells had large, slightly irregular bodies (about 6 µm diameter) (Figure 6E). The cell bodies and ducts of these cells were filled with spherical secretory granules (about 0.2 µm in diameter).

According to the CLSM images, only mucoid cells were TTX-positive (Figure 2A and Figure 5E). The toxin was uniformly distributed in perikaryon cytoplasm (Figure 5F) and ducts (Figure 5G), and it had an average intensity (Table 1).

#### 2.2.3. Integument

**Figure 7:**
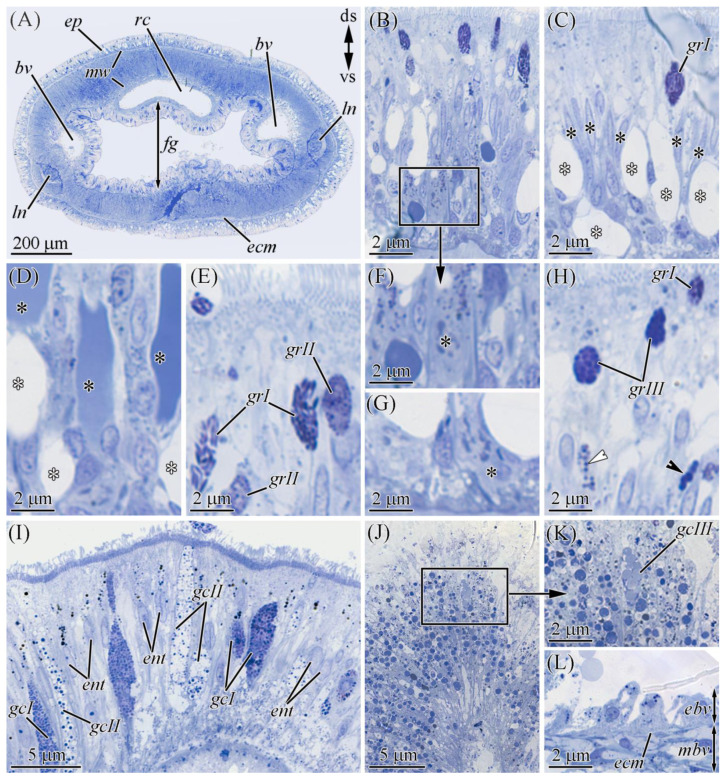
Light micrographs of the transverse sections of *Cephalothrix* cf. *simula* body in the foregut region. (**A**) Section through foregut region. (**B**) Panoramic view of the integument. (**C**) Middle and apical regions of the epidermis with multiciliated cells (black asterisks) and serous cells containing “empty” secretory granules (white asterisks). (**D**) Serous cells with full (black arrowheads) and “empty” (white arrowheads) secretory granules. (**E**) The apical region of the epidermis with apical extensions of granular cells. (**F**) The cell body of type II granular cell (asterisk). (**G**) The cell body of a type I granular cell (asterisk). (**H**) The apical region of epidermis with necks of type III and type II granular cells (black and white arrowheads, respectively), as well as apical dilations of glandular cells. (**I**) Foregut epithelium. (**J**) Epithelium of the posterior region of intestine. (**K**) Singly located apical dilation of type III glandular cell (gcIII) in the epithelium of the posterior region of intestine. (**L**) Blood-vessel wall. bv, blood vessel; ds, dorsal side; ebv, endothelium of blood vessel; ecm, extracellular matrix; ent, enterocyte; ep, epidermis; fg, foregut; gcI, type I gland cell of foregut; gcII, type II gland cell of foregut; gcIII, type III gland cell of foregut; grI, type I granular cell of integument; grII, type II granular cell of integument; grIII, type III granular cell of integument; ln, lateral nerve; mw, musculature of body wall; mbv, muscles of blood vessel; rc, rhynchocoel; vs, ventral side.

The integument of *C.* cf. *simula* was represented by pseudostratified epithelium resting on the ECM (Figure 3 and Figure 7A,B) and included one type of multiciliated cell and four types of glandular cells (one type of serous cell and three types of granular cells). The different cell types of the integument were relatively evenly interspersed in different body regions, excluding the precerebral region, where the epidermis consisted of only multiciliary cells. The ciliary cells were slender and elongated with an expanded apical region, gradually tapering to the base (Figure 7C). The apical surface of the cells contained many cilia and microvilli (Figure 2, Figure 5H–J, and Figure 7B). Serous cells were the most abundant glandular cells of the integumentary epithelium (Figure 7D). They were observed to be oval-shaped, with a single secretory granule occupying all the cell volume. A few serous cells (about 10% of all serous cells) had “empty” granules, which may be associated with the complete release of their contents during secretion on the apical surface of the integument (Figure 7B–D). Type I granular (bacillar [20]) cells had an expanded oval body (Figure 7G) with a narrower projection (neck) that distally extended to the surface of the epithelium and formed a bulb-shaped protrusion rising to the epithelium (Figure 7E,H). These cells were filled with rod-shaped secretory granules (up to 2.5 µm in length and up to 0.5 µm in diameter) (Figure 7E,H). Type II granular cells had enlarged oval bodies with proximally extended necks. In the apical region of the epithelium, the neck was expanded and protruded onto the surface of the epithelium, forming an oval-shaped papilla (Figure 7F). The whole cell volume was filled with spherical secretory granules (about 0.3 µm in diameter) (Figure 7E,H). Type III granular cells were rare, and we only identified their apical extensions (Figure 7H), which were oval-shaped and filled with spherical secretory granules up to 0.7 μm in diameter (Figure 7H).

In the immunofluorescence-stained sections of the integument of *C*. cf. *simula*, TTX was localized on the surface of the epithelium in multiciliated, serous, and type III granular cells (Figure 3 and Figure 5H–K; Table 1). In multiciliated cells, a TTX-positive antigen–antibody response was observed in the apical cytoplasm, with the toxin gradually decreasing from the distal to the proximal part of the cell, as well as in microvilli (Figure 5I,J). In serous cells, the toxin was evenly distributed within the granule and possessed a high intensity (Figure 5H,I). The apical dilutions of type III granular cells were filled with TTX-positive granules with highly intensive labeling (Figure 5J). In the basal part of the integumentary epithelium, the bodies of glandular cells with TTX-positive filamentous structures were revealed. These structures formed a network around the nucleus and partially protruded into the distal cytoplasm of the cell body (Figure 5K). It was not possible to determine to what type of glandular cell the TTX-positive bodies belonged.

#### 2.2.4. Digestive System

The digestive system consists of the post-cerebral mouth, foregut, intestine, and anus. Both the foregut and intestine epithelia of *C*. cf. *simula* consist of multiciliated phagocytic enterocytes and glandular cells (Figure 2 and Figure 7I). Two types of glandular cells were abundant and occurred in the foregut. Type I glandular cells were characterized by spherical granules (about 0.2 µm in diameter). Type II cells contained granules (about 0.4 µm in diameter) with heterogeneous contents (a centrally located dense core surrounded by a clear material) (Figure 7I). The type III cells were rare and localized in the intestine; they were characterized by spherical granules (up to 0.5 µm in diameter) (Figure 7J,K). The enterocytes were elongated and column-shaped (Figure 7I–K). The apical surface of the enterocytes contained many cilia and microvilli. The main intracellular components of these cells were phagosomes, the content and size of which from the apical to the basal part of the enterocytes were distinguished: in the apical region, they mainly had a yellow–brown (lipofuscin-like) pigment and were up to 0.5 μm in diameter; in the middle part of the cell, the phagosomes stained dark blue with methylene blue were up to 0.5 μm in diameter; and at the base of the cells, they were rather small (up to 0.2 µm in diameter), with a secretion stained from light blue to blue (Figure 7I–K). In different parts of the digestive tract, the enterocyte system only differed in the number of phagosomes: in the foregut, the number was 82 ± 20 (n = 7) on an epithelial area of 100 μm^2^, and in the anterior and posterior regions of intestine, the numbers were 324 ± 87 (n = 7) and 266 ± 34 (n = 7) on an epithelial area of 100 μm^2^, respectively.

**Figure 8:**
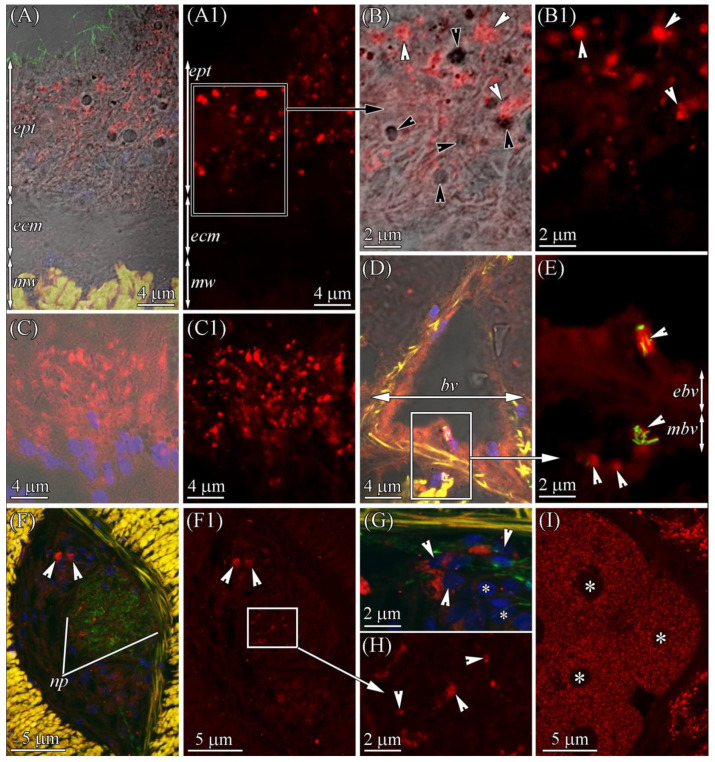
TTX-like immunoreactivity in the foregut (**A**–**B1**, **D**–**H**) and intestine (**C**, **C1**, **I**) regions of *Cephalothrix* cf. *simula*. The confocal laser scanning micrographs show substacks of transverse sections. Red, TTX-like immunoreactivity; green, α-acetylated tubulin immunoreactivity; blue, nuclei, (DAPI); yellow, musculature, phalloidin-positive. (**A**) Transmission image of intestinal epithelium (ept) with underlying body-wall muscles (mw) of the body wall united with immunostaining. (**B**) Transmission image of the intestinal epithelium united with TTX-positive (white arrowheads) and TTX-negative (black arrowheads) phagosomes. (**C**) Transmission image of intestinal epithelium in anterior intestinal region. (**D**) Transmission image of the lateral blood vessel united with immunostaining. (**E**) Terminal organ of protonephridium (arrowheads). (**F**) The lateral nerve with TTX-positive bodies of nerve cells (perikaryons) (arrowheads). (**G**) TTX-positive (arrowheads) and TTX-negative (asterisks) perikaryons of nerve cells (arrowheads). (**H**) TTX-positive nerve trunks (arrowheads). (**I**) Oocytes (asterisks). bv, blood vessel; ebv, epithelium of blood vessel; ecm, extracellular matrix; ept, epithelium; mw, musculature of body wall; mbv, muscles of blood vessel; np, neuropil.

Anti-TTX antibody staining only revealed the presence of the toxin in the enterocytes (Figure 3 and Figure 8A,B; Table 1). TTX-positive enterocytes were detected in all three parts of the digestive system (Figure 2B–D). The toxin was revealed in the cytoplasm and phagosomes of enterocytes. TTX-positive phagosomes were intensely labeled and evenly spread throughout the cell (Figure 8A–B1). Using a combination of a transmitted light detector and a fluorescence detector, we revealed that only 50% of the enterocyte phagosomes were TTX-positive (Figure 8B). In the cytoplasm, the intensity of the TTX labeling increased from the apical region of the cell to its base.

#### 2.2.5. Blood Vascular and Excretory Systems

The vascular system *C*. cf. *simula* was represented by two lateral blood vessels running along the body (Figure 2B–D and Figure 7A) and connected in the head region to form a cephalic blood lacuna. The blood-vessel wall consisting of the endothelium are rested on the ECM and 3–5 layers of longitudinal and circular muscle fibers (Figure 7L and Figure 8D). In representatives of the genus *Cephalothrix*, the excretory systems were represented by nephridia (also known as protonephridia), of which the proximal parts extend into the blood vessel (mushroom-shaped terminal organ) and the distal part forms a duct that opens on the surface of the worm’s body (see [21]). Immunohistochemical studies revealed a diffuse TTX labeling of low intensity in the cytoplasm of blood vessel endotheliocytes and the musculature lining of the blood vessels, as well as an intense TTX labeling in the cytoplasm of cells of the terminal organ of protonephridium (Figure 8D,E).

#### 2.2.6. Nervous System

The central nervous system of *C*. cf. *simula* was represented by cerebral ganglia and two lateral nerve cords (Figure 2B–D and Figure 7A). The cell bodies of lateral nerve cords lay outside the neuropil (Figure 8F,G). According to immunohistochemical studies with anti-TTX antibodies, medium-intensity labels were detected in the bodies (perikaryons) of some nerve cells and the neuropil (Figure 8F–H). In the perikaryon, TTX-positive structures looked like a net of filamentous material surrounding the nucleus and filled the perinuclear area. In the neuropil, the inner content of the nerves contained TTX-positive granules (about 0.2 μm in diameter) (Figure 8F,H).

#### 2.2.7. Reproductive System

In the reproductive system, the toxin was only detected in the cytoplasm of the oocytes (Figure 8I), which was filled with TTX-positive yolk granules of moderate intensity.

#### 2.2.8. Muscles

In the body wall, proboscis, and blood vessels, the muscles possessed weak TTX-positive labeling (Figure 5D and Figure 8D–F).

## 3. Discussion

**Figure 9:**
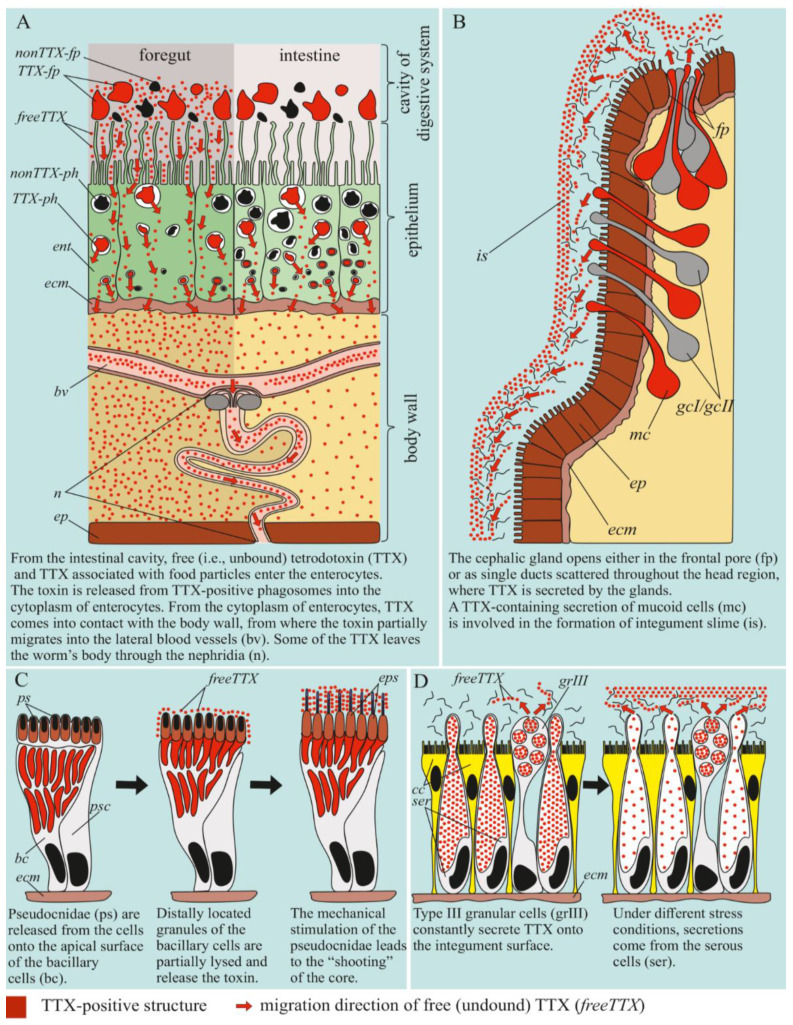
Schematic diagrams illustrating the use of tetrodotoxin (TTX) by a *Cephalothrix* cf. *simula*. (**A**) TTX uptake by the digestive system. (**B**) Constant release of the toxin by the cephalic gland. (**C**) Use of the toxin by the glandular cells of the proboscis. (**D**) Use of the toxin by the integumentary cells of the body wall. bc, bacillary cell (type II glandular cell); bv, blood vessel; cc, ciliary cell; ecm, extracellular matrix; ent, enterocyte; ep, epidermis; eps, extruded pseudocnida; FreeTTX, free TTX; fp, frontal pore; gcI/gcII, type I/II gland cell; grIII, type III granular cell; is, integumentary slime; mc, mucoid cell; nonTTX-fp, non TTX-positive food particle; nonTTX-ph, non TTX-positive phagosome; pn, protonephridial system; ps, pseudocnida; psc, pseudocnidae-containing cell; ser, serous cell; TTX-fp, TTX-positive food particle; TTX-ph, TTX-positive phagosome.

Tanu et al. [16] first demonstrated the mechanism of TTX absorption from prey tissues into a nemertean body through the phagocytic activity of intestinal cells for *Cephalothrix* sp. The authors showed that only intestinal enterocytes (“ciliary cells” according to Tanu et al. [16] or “columnar cells” according to Gibson [22]) contained TTX-positive structures, which were remarkably localized basally in cells. We found that TTX-positive phagosomes were detected in the enterocytes in all regions. Together with the TTX associated with the phagosomes, free (or unbound) toxin was found in the enterocyte cytoplasm. According to Jennings and Gibson [23], nemerteans of the genus *Cephalothrix* have a macrophage-type diet in which digestion can be divided into two almost-simultaneous phases: the first is the breakdown of food by enzymes in the foregut cavity in an acidic environment, and the second is the phagocytosis of food particles by enterocytes in various parts of the digestive system. Our data showed that, in the intestine, the numbers of phagosomes per enterocyte were about 3–4 times greater than those in the foregut. At the same time, the number of glandular cells (participating in enzyme secretion) and their diversity were greater in the foregut than in other digestive regions (Figure 3). Therefore, the main processes of food absorption through phagocytosis occurred in the intestine. Vlasenko and Magarlamov [15] found that the concentration of TTXs in the foregut region (up to 2.87 μg/g of extract) was significantly higher than that in the intestinal region (up to 0.2 μg/g of extract in middle intestine). This suggests that the foregut is the main region of toxin absorption. Since the enterocytes of the foregut region contain a relatively small number of TTX-positive phagosomes compared to the intestine, we assume that free TTX extracted from the lysed tissues of a victim in the foregut cavity is directly absorbed by enterocytes without the participation of phagosomes (Figure 9A).

Data on the distribution of the toxin in TTX-bearing animals have indicated that the toxin migrates from the digestive system to other organs through the circulatory and lymphatic systems [24,25,26] and is then excreted through specialized organs or accumulated in target organs. The role of the circulatory system in the migration of TTX was demonstrated in the puffer fish. The concentration of TTX after intravenous administration to the non-toxic puffer fish *Takifugu rubripes* gradually decreased in the blood and mainly accumulated in the skin and liver within 12 h of administration [27]. In feeding experiments with *T. rubripes*, TTX entered the blood system from the digestive tract and was then transported throughout the body, settling in the liver [27,28]. Immunohistochemical studies, including ours, have shown that nemerteans from the genus *Cephalothrix* have TTX-positive labeling in the circulatory and excretory systems [16]. These results indicate that, in nemerteans, blood is involved in the transfer of TTX and any excess is removed by the protonephridial system. However, in *C*. cf. *simula*, the toxin is distributed very unevenly; most is found in the anterior region and in the proboscis, while the concentration of TTXs in the posterior and middle regions of the body is much lower [15]. It would seem that, in the presence of a vascular blood system, the toxin should be evenly transported from the foregut and intestine and distributed throughout the nemertean body. However, as Gibson noted, only the organs of the anterior (cephalic) part of nemerteans are abundantly supplied with blood due to the branched and voluminous vascular system [22]; in nemerteans of the genus *Cephalothrix*, an additional vessel in the proboscis system was also noted [29]. In the rest of the body, blood is only carried by two lateral and narrow vessels. Thus, the specific organization of the circulatory system leads to an abundance of TTX in the head region and the proboscis.

TTX has been found in various organs and tissues of TTX-bearing animals; however, theories about the specialized functions of the toxin in organisms mainly concern the integument, venom glands, reproductive system, and “hunting” organs. TTX-containing mucus secreted by the integument of TTX-bearing animals (e.g., anemones [30] and certain fish species [31]) protects them from predators. In some cases, the secretion of the toxin into the environment is thought to perform a communication function within a population [32]. TTX also increases the survival rates for eggs and fry by making them unattractive to predators [32]. Extremely high concentrations have been found in the hunting organs of some toxic animals, e.g., in the secretions of the venomous glands of blue-ringed octopuses [33] and the extracts of the nemertean proboscis [12,15]. We found TTX-like compounds in the glands forming the cutaneous mucus of the integument and the venomous secretion of the proboscis (the nemertean hunting organ), as well as in the cytoplasm of eggs, which suggested that nemerteans use the toxin to repel predators, protect offspring, and immobilize prey.

The nemertean integument was found to be represented by the glandular epithelium, which forms abundant mucus (slime) that envelops the animal [20]. The subepidermal cephalic gland in the anterior end of nemerteans forms ducts through which secretions are released onto the surface of the worm [34]. The epidermal mucus performs many functions associated with the mechanical protection of the soft worm body against solid particles. It facilitates sliding, bearing offspring, forming “houses” [20,35,36], and scaring off predators [5,12,15]. In the current study, we found that, of the four types of glandular cells in the integument of *C*. cf. *simula*, only two contained TTX-positive secretions: serous and type III granular cells. In the cephalic gland, the label was only found in mucoid cells. Serous cells are the most abundant type of epidermal glands and quickly secrete [20,36], which probably explain the “empty” serous cells found in the integument (Figure 7C,D). Several authors believe that such an instant release serves to quickly form a slime “house” and serves as a defense mechanism under stressful conditions [5,35]. Other ectodermal glandular cells (including the granular cells of the integument and cells of the cephalic gland) form a constant background in the slime of nemerteans, so the secretory granules of the granular cells of the integument are released individually by exocytosis [20,36]. The cells of the cephalic gland produce mucus, which is released onto the surface of the epidermis [19]; that is, in *C*. cf. *simula*, TTX-positive integument granular cells and TTX-positive mucoid glandular cells of the cephalic gland can maintain a constant concentration of the toxin in the slime surrounding the worm.

Nemerteans are predominantly predators, usually using an everted proboscis as a weapon. Several studies have shown that the proboscis secretes a sticky venom that contains numerous lytic and/or toxic substances [12,37,38]. In the current study, we revealed the presence of TTX-like compounds in the granules of bacillary cells and a secretion accumulating in the lumen of the proboscis. The bacillary cells were associated in pairs with pseudocnid-containing cells. Such a glandular system has also been described for other representatives of Palaeonemertea and Pilidiophora nemerteans [18,39,40], and, according to the hypothesis of Montalvo et al. [39], this system may play a role in prey retention; that is, the “sticky” component of the bacillary cell secretion can enhance pseudocnidae’s adhesion to the surface of a victim’s body while the toxic component immobilizes it (current research).

In a study of the TTX distribution in nemertean *Kulikovia alborostrata* (also known as *Lineus alborostratus*) by Magarlamov et al. [9], a possible pathway for the migration of the intracellular toxin was proposed. The sites of intracellular toxin localization were shown to be secretory granules of certain cell types and their synthetic apparatus. The authors suggested that, upon entering the cell, TTX was deposited in the cistern of the endoplasmic reticulum and Golgi bodies and then bound to its tropic structures, probably proteins. Then, the bound toxin transferred to the secretory granules. The results of this study indicated a similar intracellular localization of TTX in *C*. cf. *simula*. It is worth noting that the TTX-binding transport proteins were first discovered in puffer fish [41], and their presence in other animal species bearing TTX was assumed [42]. The existence of such substances in nemerteans should be clarified, and this could be a perspective direction for further research.

## 4. Materials and Methods

Representatives of *C*. cf. *simula and K. alborostrata* were collected in the rhizoids of the brown algae *Saccharina* sp. in Spokoinaya Bay, Peter the Great Bay, in the Sea of Japan (42.7090° N, 133.1809° E) in August 2020. Nemertean species were identified by Dr. Alexey V. Chernyshev, an expert on nemertean zoology. The worms were kept in aerated aquariums with running seawater for 24 h at 17 °C. The specimens were anesthetized in a 7% solution of magnesium chloride, and several segments were cut off from each worm: the proboscis, the precerebral (head), the foregut, and the anterior and posterior intestines. For immunohistochemical studies, the samples were fixed in 4% paraformaldehyde in phosphate-buffered saline (PBS), pH 7.4, for 1.5 h, washed three times with PBS for 15 min at room temperature, placed in a 20% sucrose solution, and incubated for 24 h at 4 °C. After that, the samples were placed in a Leica OCT cryocompound tissue freezing medium solution (Leica, Germany), and 10 μm thick cryosections were made using a Thermo HM 560 microtome (Thermo Fisher Scientific, Waltham, MA, USA). The cryosections were washed in PBS with Tween-20 (PBST) and then incubated in a 1% Triton 100× solution in PBS for 1 h at room temperature. The sections were washed in PBS and placed in a blocking solution of 5% bovine serum albumin (BSA) in PBS for one day at 4 °C. The following primary antibodies were used: mouse anti-acetylated-α-tubulin antibodies (Sigma-Aldrich, St. Louis, MO, USA) (dilution 1:1000) and rabbit anti-TTX antibodies (Genetex, Irvine, CA, USA) (dilution 1:25). The samples were incubated with a mixture of primary antibodies for two days at 4 °C, washed in PBST and incubated overnight at 4 °C in a mixture of Alexa 488 Goat Anti-Mouse secondary antibodies (Invitrogen, Waltham, MA, USA) (dilution 1:500) and Alexa 647 Goat Anti-Rabbit antibodies (Invitrogen, Waltham, MA, USA) (dilution 1:500). The sections were washed with PBS and counterstained with 4′,6-diamidino-2-phenylindole (DAPI) and phalloidin Alexa Fluor 546 (Thermo Fisher Scientific, Waltham, MA, USA). The processed slices were embedded in Mowiol 4-88 (Sigma-Aldrich, St. Louis, MO, USA), mounted on glass slides, and analyzed on an LSM-780 (Carl Zeiss, Germany). Both non-immune rabbit serum and PBS with 10% bovine serum albumin were used as negative controls, and no positive reactions were observed in any of the examined sections. The specimens were also viewed under a 647 filter to rule out aberrant autofluorescence. Cryosections of *K. alborostrata* were used as positive controls (see Supplementary File S1 and S2).

For morphological studies, the material was fixed in a 2.5% glutaraldehyde solution in PBS for 1 h, followed by washing in PBS. Post-fixation was carried out in a 1% OsO_4_ solution for 1 h. Then, the material was dehydrated in ethyl alcohol and acetone and embedded in epon–araldite resin (EMS, Hatfield, PA, USA). Transverse semithin sections (1 μm thick) were made with an Ultracut E ultramicrotome (Reichert, Germany) and stained with methylene blue (Sigma-Aldrich, St. Louis, MO, USA). The material was analyzed using a Zeiss Axio Imager 2 microscope (Zeiss, Germany). Adobe Photoshop 2019 (Adobe, San Jose, CA, USA) was used for image processing.

For TTX identification and quantification, chromatographic methods were used. The extracts were obtained according to the protocol described by Vlasenko et al. [10]. The identification and quantification of TTX were performed using high-performance liquid chromatography with tandem mass spectrometry (HPLC–MS/MS) according to Bane et al. [43] with modifications described by Vlasenko et al. [10].

The intensity of the fluorescent label was estimated using Adobe Photoshop 2019 (Adobe, San Jose, CA, USA). The intensity of the label was evaluated by calculating the average pixel brightness for the selected area. In this case, the brightness of the mark in the muscles was taken as low intensity, and the brightness of the mark in the phagosomes of intestinal enterocytes was taken as high intensity. Raw data are available at Figshare (https://figshare.com/, accessed on 19 August 2021): doi:10.6084/m9.figshare.15180180. 

## Figures and Tables

**Table 1:** Tetrodotoxin (TTX)-like-positive structures in different organs and tissues of *Cephalothrix* cf. *simula*.

Organ/Tissue	Intracellular Structure	Intensity
Proboscis	Secretory granules of type II glandular cells	+++
	Myocytes	+
	Perinuclear area of type II glandular cells	++
	Glandular epithelium surface and proboscis lumen	+
Cephalic gland	Body and duct of mucoid cells	++
Epidermis	Secretory granule of serous cells	+++
	Secretory granules of type I granular cells	+++
	Perinuclear area of secretory cells	++
	Microvilli of ciliary cells	+++
	Cytoplasm of ciliary cells	+
Digestive system	Phagosomes of enterocytes	+++
	Cytoplasm of enterocytes	++
Blood vessels	Cytoplasm of endotheliocytes	+
	Myocytes	+
Excretory system	Terminal cells of protonephridium	+++
Lateral nerves	Neuropil	++
	Cytoplasm of perikaryon	++
Oocyte	Yolk granules	++
Body wall	Musculature	+

+ weak TTX-like immunoreactivity; ++ medium TTX-like immunoreactivity; +++ high TTX-like immunoreactivity.

## Supplementary Materials

The following are available online at https://www.mdpi.com/article/10.3390/md19090494/s1, Supplementary File S1, Figure S1: TTX-like immunoreactivity of the integument of heteronemertea *Kulikovia alborostrata*; Supplementary File S2, Figure S2: TTX standard, Figure S3: TTX of *K.alborostrata*.
