# Supplementary material for: Intrabody Tetrodotoxin Distribution and Possible Hypothesis for Its Migration in Ribbon Worms Cephalothrix cf. simula (Palaeonemertea, Nemertea)
Source: Mar Drugs. 2021 Aug 29;19(9):494. doi: 10.3390/md19090494 (PMC8465930; doi:10.3390/md19090494)
Supplement: Supplementary file 1 [file marinedrugs-19-00494-s001.zip › Supplementary S1.pdf]

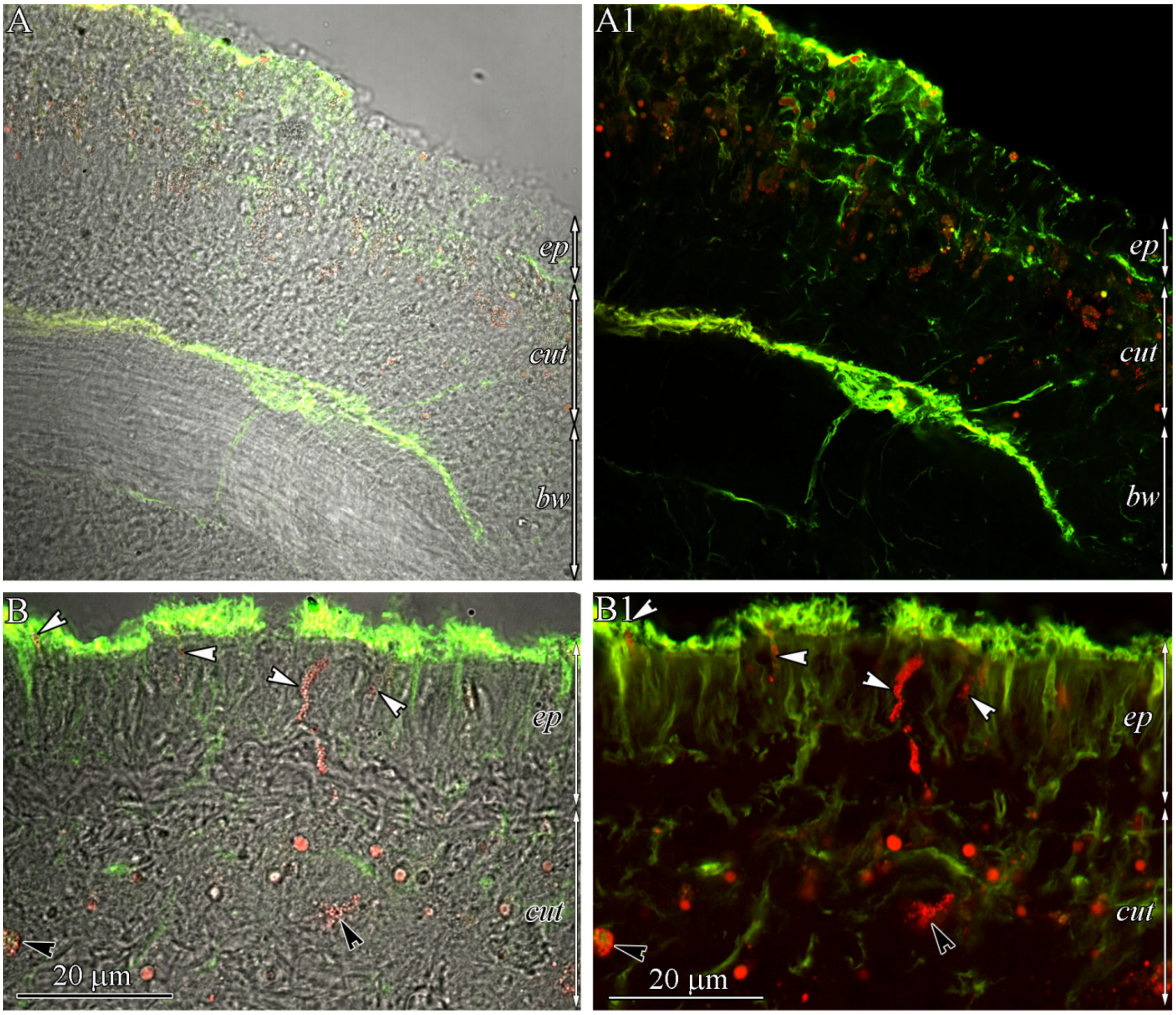

Figure S1. TTX-like immunoreactivity of the integument of heteronemertea *Kulikovia alborostrata*. Confocal laser scanning micrographs, substacks of transverse sections. Red, TTX-like immunoreactivity; green, α-acetylated tubulin immunoreactivity; blue, nuclei (DAPI). (A) Panoramic view showing body wall (B) The epidermis (ep) contains apical extension (white arrowheads) of bacillary gland cells type I that are filled by TTX-positive granules; the cutis (cut) (subepidermal layer) contains bodies of bacillary gland cells type I that are filled by TTX-positive granules (black arrowheads). bw, body wall; ep, epidermis, cut, cutis
